# Retraction Note: Norcantharidin combined with Coix seed oil synergistically induces apoptosis and inhibits hepatocellular carcinoma growth by downregulating regulatory T cells accumulation

**DOI:** 10.1038/s41598-024-57766-9

**Published:** 2024-03-26

**Authors:** Dan Wang, Chendong Yang, Zhuien Wang, Yi Yang, Defang Li, Xiaojie Ding, Wenjuan Xu, Qiusheng Zheng

**Affiliations:** 1 Binzhou Medical University, Yantai, 264003 China; 2 Yantai Hospital of Traditional Chinese Medicine, Yantai, 264003 China

Retraction of: *Scientific Reports* 10.1038/s41598-017-09668-2, published online 24 August 2017

The Editors have retracted this Article. After publication, concerns were raised regarding the images presented in the figures, specifically:

- Figure 2a HepG2 images appear highly similar to Fig. 1b of^1^;

- Figure 2b HepG2 Control and NCTD 0 h images appear highly similar;

- Figure 3d HepG2/ADM b-actin appears highly similar to lanes 2–5 of Fig. 5c b-actin;

- Figure 7d CTLA-4 appears highly similar to PGAM5 in Fig. 6b of^2^.

The Authors have provided the underlying raw data, which suggest that the colony formation experiment results in Fig. 2a were based one well per group, and a number of wound healing images provided for Fig. 2b representing different repeats appear to originate from the same samples. Additionally, Fig. 5c FoxP3 and b-actin blots appear to be differently spliced.

The Editors therefore no longer have confidence in the presented data.

Wenjuan Xu agrees to this retraction. Defang Li and Qiusheng Zheng have not responded to any correspondence from the Publisher about this retraction. The Publisher has not been able to obtain current email addresses for Dan Wang, Chendong Yang, Zhuien Wang, Yi Yang and Xiaojie Ding.
